# Do social groups prevent Allee effect related extinctions?: The case of wild dogs

**DOI:** 10.1186/1742-9994-10-11

**Published:** 2013-03-15

**Authors:** Elena Angulo, Greg S A Rasmussen, David W Macdonald, Franck Courchamp

**Affiliations:** 1 Ecologie, Systématique & Evolution, UMR CNRS 8079, University Paris Sud, Orsay Cedex, 91405, France; 2 Estación Biológica de Doñana, CSIC, Avda. Americo Vespucio s/n, Sevilla, 41092, Spain; 3 Wildlife Conservation Research Unit, Zoology Department, University of Oxford, The Recanati-Kaplan Centre, Tubney House, Tubney, Oxfordshire, OX13 5QL, UK

**Keywords:** Group augmentation, Group size, *Lycaon pictus*, Obligate cooperative breeding, Positive density dependence

## Abstract

**Background:**

Allee effects may arise as the number of individuals decreases, thereby reducing opportunities for cooperation and constraining individual fitness, which can lead to population decrease and extinction. Obligate cooperative breeders rely on a minimum group size to subsist and are thus expected to be particularly susceptible to Allee effects. Although Allee effects in some components of the fitness of cooperative breeders have been detected, empirical confirmation of population extinction due to Allee effects is lacking yet. Because previous studies of cooperation have focused on Allee effects affecting individual fitness (*component* Allee effect) and population dynamics (*demographic* Allee effect), we argue that a new conceptual level of Allee effect, the *group* Allee effect, is needed to understand the special case of cooperative breeders.

**Results:**

We hypothesize that whilst individuals are vulnerable to Allee effects, the group could act as a buffer against population extinction if: (i) individual fitness and group fate depend on group size but not on population size and (ii) group size is independent of population size (that is, at any population size, populations comprise both large and small groups). We found that both conditions apply for the African wild dog, *Lycaon pictus*, and data on this species in Zimbabwe support our hypothesis.

**Conclusions:**

The importance of groups in obligate cooperative breeders needs to be accounted for within the Allee effect framework, through a group Allee effect, because the group mediates the relationship between individual fitness and population performance. Whilst sociality is associated with a high probability of Allee effects, we suggest that cooperative individuals organized in relatively autonomous groups within populations might be behaving in ways that diminish extinction risks caused by Allee effects. This study opens new avenues to a better understanding of the role of the evolution of group-living on the probability of extinction faced by social species.

## Introduction

### Cooperative behaviour and Allee effects

Cooperative behaviour, how it evolved and why it persists, are fundamental questions in the theory of natural selection [1]. Early explanations of cooperative behaviour were formulated in the mid-XX^th^ century and based on benefits to groups and populations [2,3]. While these authors did not offer evolutionary explanations [4], Allee proposed a theoretical explanation of collaboration at the population level, now known as the Allee effect [2]. The Allee effect states that, in some species, individual fitness at low population numbers shows positive density dependence: the greater their number, the better their fitness. Thus, for cooperative species, as the number of individuals decreases, the collective benefits of cooperation may diminish disproportionately and, consequently, one or more components of individual fitness may decrease. The Allee effect, also called positive density dependence, does not exclude that the effects of competition lead to negative density dependence at high densities. Although, both, Allee effects and negative density dependence can be present in the population dynamics of a given species [5], here we focus at processes of population dynamics occurring at low population densities such as the ones resulting from the benefits of cooperation.

Empirical investigations of the theory of Allee effects were rare and scattered until the end of the 1990’s when three papers emphasised the importance of Allee’s ideas for the conservation of endangered species [5-7]. Since then, most literature on Allee effects distinguish between different individual fitness components, and argue that the relative strength of the Allee effects affecting different individual fitness components will determine the effects on the population e.g. [7-13]. This distinction follows Stephens et al. [7], who suggested it would be helpful to differentiate between component Allee effects (Allee effects manifested by a component of individual fitness, e.g. benefit in terms of increased survival or reproductive rates when numbers increase) and demographic Allee effects (Allee effects manifested at the level of total fitness by a lower *per capita* population growth rate at lower numbers). Thus, for a given species, one component Allee effect (e.g., survival) could be cancelled out by negative density dependence in another (e.g., reproduction), with the net result of no demographic Allee effect [7,8]. Alternatively, demographic Allee effects may be generated by the addition of two or more component Allee effects [8]. When the demographic Allee effect is strong, it may drive the population into a feedback loop of exacerbated likelihood of extinction [5,6,8].

Evidence of component Allee effects is starting to accumulate but, surprisingly, empirical demonstrations of demographic Allee effects remain very few [8,12,14]. There are many mechanisms by which we can predict which groups of species might be especially prone to Allee effects (e.g. [8,9]). From the point of view of cooperation, the Allee effect theory states that the more the individuals of a species need to cooperate, the more intense Allee effects are expected in that species. This is because the individual benefits coming from cooperation decrease when the number of individuals decreases. Thus, the absence of empirical evidence of demographic Allee effects is most noticeable for cooperative breeding species, where Allee effects are most expected.

The Allee effect, as a theoretical construct, is clear-cut at the individual and at the population levels in non-social species. However, it is unclear how these effects, and their ultimate consequence, population extinction, may operate in group-living species. For cooperative species, the link between individual fitness and population demography is mediated by the dynamics of the groups, which may be subject to Allee effects; but evidence for demographic Allee effects is rare. Stephens et al. [7], attempting to resolve confusion around the Allee effect, mentioned that some ‘inconsistencies’ may occur between the fitness within the group and at a wider scale. In this article, we first present a hypothesis of how Allee effects could act in obligate cooperative breeding species, by linking the existing understanding of the evolution of living in groups with the theory of Allee effects. In order to do that, we propose to introduce the ‘group Allee effect’ a necessary step to understand Allee effects in obligate cooperative breeders, distinguishing it from the component and demographic Allee effects. Next, we present an empirical analysis illustrating our argument with field data on an obligate cooperative breeder, the African wild dog, *Lycaon pictus*. This leads to an explanation of the apparent paradox of why high extinction rates are not observed amongst species theoretically prone to Allee effects.

### The hypothesis: group living, group augmentation and group Allee effects

Obligate cooperative breeding species are at the extreme of a eusociality continuum [15], where breeding is generally restricted to only a few of the potentially reproductive individuals of the group. Kinship [16] is clearly a candidate as a selective pressure in the evolution of obligate cooperative breeding, insofar as adult group members are often related [17]. However, collaboration among unrelated members in cooperatively breeding groups is not uncommon [18,19], and other selective pressures, such as the benefits incurred by group augmentation, may also favour obligate cooperative breeding [18]. The group augmentation hypothesis suggests that individuals survive or reproduce better in large groups; it is a plausible explanation for group living, alone or in concert with kin selection [18]. Multi-level selection theory – which partitions the total evolutionary response to selection into distinct between-group and within-group components – seeks to create a useful distinction between the interests of individuals and the needs of the group [4,20,21]. Because individuals of obligate cooperative breeders can survive only in a group, individual fitness and group performance (the latter accounting for group survival and growth rate) are inter-dependent, meaning they depend on group size. This means that that there should be an optimal group size or threshold below which the group is disadvantaged e.g., [22-27].

The same prediction follows from applying the concept of an Allee effect to the group, a “group Allee effect”: group performance and size are predicted to be positively related, at least up to a point. The link between performance and size of a group is not new, but in fact, we need to distinguish a group Allee effect within the Allee effect theoretical framework when talking about obligate cooperative breeders. In the same way that the structure of these species has three levels (individuals are structured in groups and the groups form the populations), the Allee effect has to take into account these three levels: the individual fitness, the group performance and the population performance. If group Allee effects were to follow the same dynamic as a component Allee effect, we would expect demographic Allee effects in group-living species, as well as their ultimate manifestations, extinction. Courchamp et al. [5,9,28] have indeed suggested that Allee effects at the group level might contribute to a demographic Allee effect: high extinction rates amongst small groups might increase the risks of population extinction.

However, we know of no report of population extinctions due to Allee effects for obligate cooperative breeders. Indeed, recent works on these species have shown populations with component and group Allee effects but consistently with no demographic Allee effect [29-32], see Table 1 and Additional file 1. The key to this conundrum may be that these obligate cooperative species may not display demographic Allee effects, precisely because of the role of groups in their population dynamics. Recently, three studies have given some insight following this line. First, Bateman et al. [30,31] not only suggests that component Allee effects may not translate to the population level but highlights that explicit consideration of population structure (group dynamics and their interaction with population-level dynamics, e.g. asynchrony of group growth rates) will be the key to understanding the mechanisms of this lack of translation for cooperatively breeding species. Second, Woodroffe [32] has recently suggested that the lack of relationship between pack size and population size was a likely explanation for the absence of translation of component Allee effects into demographic Allee effects.

**Table 1:** Detection of Allee effects (AE) and pack-population size relationships in Lycaon pictus (Y: yes and N: no Allee effect detected)

		**Courchamp et al. 2000 [**[28]**]**	**Courchamp & Macdonald 2001 [**[33]**]**	**Courchamp et al. 2002 [**[26]**]**	**Creel & Creel 2002 [**[34]**]**	**Creel et al. 2004 [**[35]**]**	**Carbone et al. 2006 [**[36]**]**	**Buettner et al. 2007 [**[37]**]**	**McNutt & Silk 2008 [**[38]**]**	**Rasmussen et al. 2008 [**[27]**]**	**Somers et al. 2008 [**[29]**]**	**Gusset & Macdonald 2010 [**[39]**]**	**Wodroffe 2011 [**[32]**]**	**This study**
Demographic AE		Y									N		N	N
Component AE	Survival pups		Y	Y	Y/N	Y			Y		N	N	N	Y
	Survival yearlings				N			Y			N	N	N	N
	Survival adults				N						N	N	N	N
	Survival dispersers										N			Y
	Dispersal group size										N			N
	Breeding - success							N					N	
	Breeding - litter size		Y		Y	Y		Y	Y	Y	N	Y	Y	Y
	Hunting - success				Y					N				
	Hunting - efficiency		Y	Y	Y		Y							
	Pup guarding			Y										
	Energetic balance									Y				
Group AE	Pack extinction	Y								Y			N	
	Pack formation	Y									Y			Y
	Life span													Y
	Growth rate													Y
Allee threshold 1		5												
Allee threshold 2				5									N	4
Pop. size vs pack size					N						Y		N	N
Country		-	-	Z	T	SAk,T,B	T	SAk	B	Z	SAh	SA	K	Z
Area (km^2^)		-	-	5500	2600	9480	-	-	-	5500	900	380	-	6000
Date range		-	-	94-99	91-96	89-03	64-87	89-04	15 y	94-02	80-04	95-06	00-08	89-02
Population trend		-	-	I	S	-	D	-	-	I	Ri	Ri	Rc	I
Pop size range (# indiv)		-	-	-	880	>700	-	-	700-986	-	3-31	-	10-200	7-53
Pack size range (# indiv)		-	-	-	3-20	6-13	-	-	2-30	-	2-24	2-17	3-21	2-15

Before 2000, literature on the effects of pack size is reviewed by Courchamp & Macdonald [33]. More information on each parameter of each paper can be found in Additional file 1. Because Creel et al. [35] studied 3 different populations, the data for the area is the sum of the three areas, but for date, population and pack size range, we took the highest range between the three populations. Allee threshold 1 refers to the number of packs in the population. Allee threshold 2 refers to the size of the pack. Countries: B: Botswana; K: Kenya; SA: South Africa (k: Kruger National Park; h:Hluhluwe-iMfolozi Park); T: Tanzania; Z: Zimbabwe. Population trend: D: decreasing, I: increasing, Ri: reintroduced, Rc: recolonization, S: stable.

These three studies have complementary suggestions focusing on the links between groups and populations but have not produced a mechanism. Consequently, we go one step further and consider links between the individual fitness and the population size. Here, we propose that because groups constitute a level of organization within their own dynamics, individual fitness may be more dependent on group size than on population size. Two lines of evidence support this proposition. First, when animals are not organized into cooperative groups there is often a direct, reciprocal relationship between individual fitness and population size [6]. Where group-level organization is interposed between the individual and the population, such reciprocity may no longer occur: the fates of individual groups may be little affected by the sum of sizes of all groups (i.e., the population size). Second, if an Allee effect results in an increase in individual fitness due to cooperation [5,7], individual fitness should be positively related to the number of cooperating individuals within a group. Thus, individual fitness should be less dependent of population size. If there is no cooperation between groups, group performance might even be independent of population size.

Taking into account all these points, we can hypothesize that if Allee effects were to act only at the individual and group levels and not to affect the population level, growth rate of a given group should be independent of a) growth rates of other groups and b) population size (at least to a certain point, as it could be that at high population size the growth rate of a group is limited by negative density dependence). Thus, the fates of groups would differ as a consequence of their size (due to group Allee effects) and small and large groups could coexist irrespective of population size (asynchrony of group growth rates, as suggested by Bateman et al. [30]). Therefore, group size could be independent of population size (as shown by Woodroffe [32]) while population size would nonetheless depend on the size (and number) of the groups. Dispersal would provide a mechanism whereby the presence of large groups would compensate for the extinction of small ones (as shown by Bateman et al. [31]). In these circumstances, group dynamics would act as a buffer, the largest packs compensating for decreases in individual fitness due to reduced cooperative benefits of the smallest packs. This would prevent component Allee effects from translating into demographic Allee effects, and thus avert population extinction. If our hypothesis is correct, the fact that cooperative breeding species live in groups makes them prone to component and group Allee effects, but the structure of the groups within the population would prevent Allee effects causing the extinction of populations. This model could contribute to the explanation of the evolution of extreme cooperative behaviour, despite a predicted increase of susceptibility to Allee effects (and thus extinction risk).

### Testing the hypothesis: example data analysis

Our core hypothesis is that, under circumstances specified below, Allee effects at the levels of both individuals and groups will be determined by group size and not by population size. We also explore what relationship exists between individual and group performances with population size, dynamics and extinction. We approach this, following the conceptual framework above, by testing (i) the existence of component and group Allee effects, dependent on group numbers and independent of population numbers; (ii) the absence of demographic Allee effect; and, (iii) the independence between group size and population size. Hereafter we use the term “group Allee effect” for any relationship between group fitness (such as group growth rate) and number of individuals (such as group or population sizes).

We used as a model species the African wild dog, *Lycaon pictus*, an endangered carnivore that has proven to be an insightful model species for the study of Allee effects; Table 2. It is an obligate cooperative breeder that lives in groups (also called packs), where breeding is generally restricted to the alpha pair, subordinate females being reproductively suppressed. Non-reproductive individuals help to raise pups and all group adults hunt cooperatively and share kills equitably [40]. Packs are usually formed when a small same-sex subgroup (usually litter-mates) leave their natal pack and join at least one subgroup (also litter-mates) of the opposite sex [41]. Once bonded they usually stay together until the end of the pack life. The growth of such packs is normally through the birth of pups, which may remain there some years in the company of the original adults (their parents, uncles and aunts). Consequently, as packs get larger, there continues to be close genetic relatedness between adults and brood as well as the original adult members of same sex. Occasional immigration of unrelated adults may occur [42] though in this study it only occurred in small packs (less than 5 individuals). A pack life ends when all individuals die or when individuals disband.

**Table 2:** **The*Lycaon*as a model species for Allee effects**

**Published studies**	**Description of the use of *Lycaon *as a model for Allee effects**
Courchamp and Macdonald 2001 [33]	Review of the literature to suggest the crucial importance of Allee effects in *Lycaon*, and discussion of different mechanisms and processes that could advantage larger packs. These included, improved hunting efficiency, defense against kleptoparasites, better ability to exploit prey species range, improved adult survival due to increased predator vigilance, higher litter size and subsequent survival of pups due to pup-guarding.
Courchamp et al. 2000 [28]	A modelling exercise showing that should those hypothesized Allee effects arise, they could cause both high rates of pack extinction and affect the colonization of new territories, ultimately increasing population extinction rates.
Courchamp et al. 2002 [26]	The first empirical test of the existence of component Allee effects. Data showed that not only did the probability of pup-guarding increase with pack size, but that there was a pack size threshold below which this vital activity for pups can not longer be systematically accomplished.
Creel and Creel 2002 [34], Creel et al. 2004 [35], Carbone et al. 2005 [36], Bluettner et al. 2007 [37], McNutt and Silk 2008 [38], Rasmussen et al. 2008 [27], Gusset and Macdonald 2010 [39]	These empirical studies have searched for Allee effects in a variety of populations (see Table 1). Earlier studies that tested relationships between fitness and individual numbers (e.g. pupguarding data) were too limited in sample size and so not analyzed.
Somers et al. 2008 [29], Woodroffe 2011 [32]	They explored effects at the population level

The same features of their natural history that make *Lycaon* likely victims of the Allee effects also make them a good model to test and understand Allee effects.

## Results

**Figure 1:**
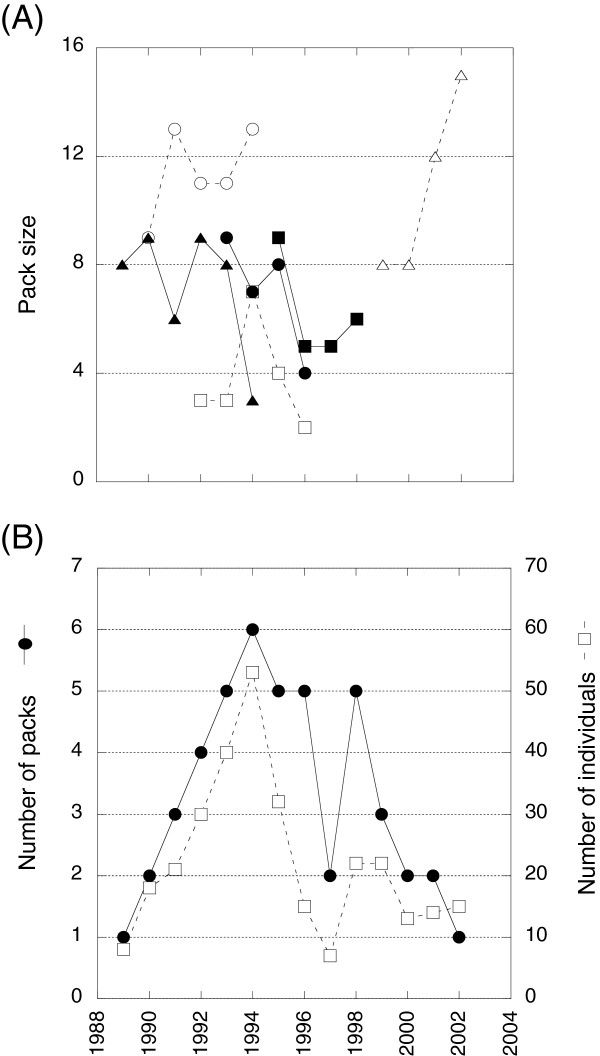
**Evolution of the *Lycaon *population during the study period.** (**A**) Pack sizes in long-life packs and (**B**) total number of packs and individuals.

In total, twenty packs were followed, although in some cases the information was incomplete for some parameters. Figure 1A displays the history of 6 packs for which we had complete information spanning > 4 years. Packs either ceased to exist because they went extinct (n = 16) or individuals disbanded (n = 2), or they survived until the end of the study period (n = 2). The *Lycaon* population within the Hwange study area included an average of 3 packs/year (ranging from 1 to 6), totaling an average of 22 individuals/year (ranging from 7 to 53) (Figure 1B).

### Individual fitness depends on group size: component Allee effect

**Figure 2:**
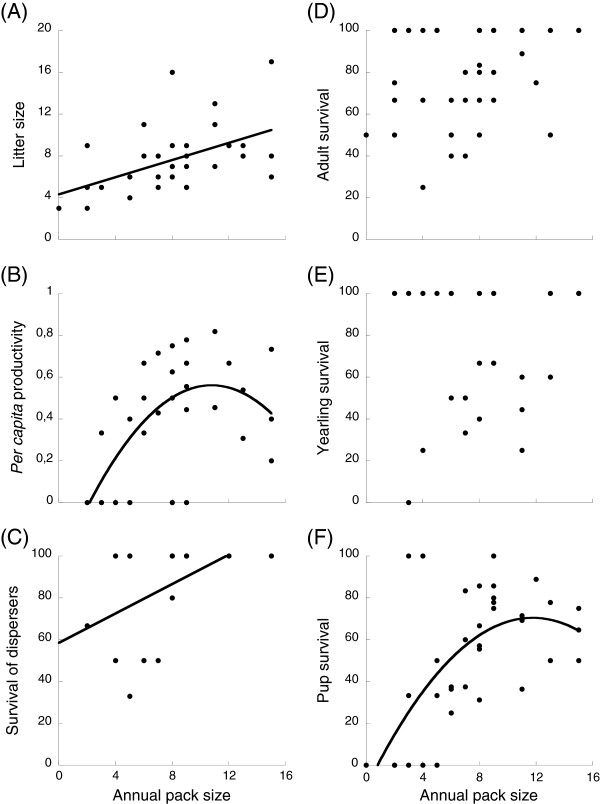
**Relationships between annual individual fitness and annual pack size in *Lycaon*.** (**A**) Litter size, (**B**) *per capita* productivity, (**C**) survival of dispersers, (**D**) survival of adults, (**E**) survival of yearlings and (**F**) survival of pups. Circles represent observed values for each pack in each year. Model fits revealing component Allee effects are shown when statistically significant. Fits correspond to a simple linear regression of raw data, not to the fits for the generalized linear models.

A general overview of the analyses for Allee effects revealed several component Allee effects caused by pack sizes but not by population sizes: some traits of individual fitness were positively related to pack sizes but none of them was correlated to population sizes (Table 3). Litter size was positively and significantly correlated with pack size, showing an Allee effect related to reproduction at lower pack sizes (Figure 2A). *Per capita* productivity, that is, the number of pups remaining at the end of the yearly period divided by pack size, was also positively correlated to pack size at small pack sizes, demonstrating an Allee effect; and negatively related to pack sizes at high sizes demonstrating negative density dependence at those sizes. Smaller packs had lower *per capita* productivity than did packs of medium sizes (Figure 2B). Using the estimated quadratic fit, the pack size that maximized the *per capita* productivity was 10.20 individuals. Similarly, pup survival (survival since birth to the next breeding period) was significantly lower in smaller packs and higher at medium pack sizes (Figure 2F). In this case the pack size that maximized pup survival was 11.76 individuals. Survival of dispersers (survival since the start of the dispersion event to the next breeding season) followed the same trend as did litter size showing a survival-related component Allee effect: survival of dispersers was significantly lower in smaller packs (Figure 2C). However, there was no such relationship for either adult or yearling annual survival rates (Figure 2D, E). Larger packs did not produce larger groups of dispersers in this population (χ^2^ = 0.82, *P* = 0.36, n = 17).

**Table 3:** **Effects of*Lycaon*pack size and population size on demographic traits**

**Abundance (x) ⇒**			**Pack size**						**Population size**			
**Fitness trait (y) ⇓**	**R**	**E**	**N**_**1**_	**N**_**2**_	**χ**^**2**^	**P**	**fit (y =)**	**SE**	**N**_**1**_	**N**_**2**_	**χ**^**2**^	**P**
Litter size	Y	N	34	16	**4.49**	**0.034**	4.328 + 0.411x	0.857, 0.110	34	16	3.49	0.062
*Per Capita* productivity	Y	N	46	16	**7.60**	**0.006**	– 0.317 + 0.163x	0.090, 0.027	46	16	2.42	0.120
					**6.42**	**0.011**	- 0.008x^2^	0.002				
Survival of adults	Y	B	47	17	1.55	0.214			47	17	0.00	0.981
Survival of yearlings	Y	B	25	12	0.55	0.457			25	12	0.94	0.332
Survival of pups	Y	B	38	17	**8.83**	**0.003**	- 4.515 + 0.931x	1.005, 0.198	38	17	1.17	0.280
					**7.28**	**0.007**	- 0.040 x^2^	0.009				
Survival of dispersers	Y	B	17	9	**4.38**	**0.036**	- 0.873 + 0.346x	0.978, 0.117	21	11	0.18	0.673
Pack growth rate	Y	N	34	16	**4.73**	**0.030**	0.115 + 0.266x	0.147, 0.044	34	16	0.95	0.329
					**4.55**	**0.033**	- 0.013x^2^	0.002				
Pack life span	N	N		18	**11.96**	**<0.001**	11.467 + 3.874x	6.099, 0.940		18		
Pack formation*	N	N		13	**8.99**	**0.003**	- 0.630 + 0.409x	0.598, 0.114		13	1.03	0.311
Population growth rate**	N	N								12	2.39	0.122

R indicates whether the model has a repeated measure term or not (Y: yes, N: no). E indicates the type of distribution of errors of the models (N = normal, B: binomial). N is the sample size: the number of packs per year (N_1_) and the number of different packs (N_2_). Chi-square (χ^2^) and P are statistics for the GLM performed – in bold when significant. Fits are shown only for statistically significant models, with the standard errors (SE) of the estimates. * Pack formation is tested against the number of packs in the population instead of the pack size. **Sample size corresponds to the number of years of monitoring (from 1990 to 2002).

### Group performance depends on group size and numbers: group Allee effect

**Figure 3:**
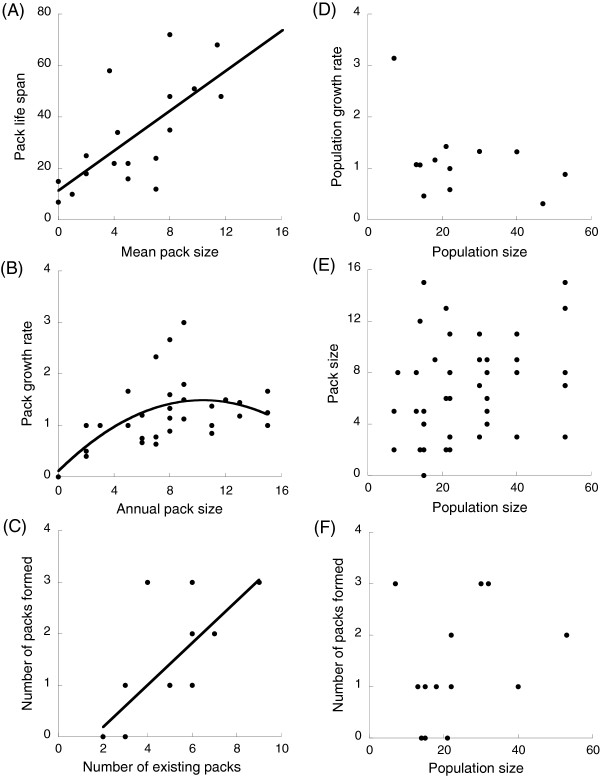
**Relationships between pack and population performance and pack and population size in Lycaon.** (**A**) pack life span in months, (**B**) annual *per capita* pack growth rate, (**C,F**) number of pack creation events per year, (**D**) annual *per capita* population growth rate and (**E**) annual pack size. Circles represent observed values for each pack (**A**), for each pack in each year (**B, D**) and for each year (**C**); model fits revealing group and demographic Allee effects are shown when statistically significant. Fits correspond to a simple linear regression of raw data, not to the fits for the generalized linear models.

We also found several group Allee effects (Table 3), showing that the above component Allee effects have consequences at the pack level. There was a significant, positive relationship between mean pack size and pack life span: smaller packs had the shortest life span (Figure 3A). There was also a quadratic, significant relationship between annual pack size and annual *per capita* pack growth rates: smaller packs had a slower growth rate than did medium-sized packs (Figure 3B). Using the estimated fit for the quadratic relationship between *per capita* pack growth rate and pack size (see Table 3), we estimated that pack growth rate was positive at pack size equal to, or higher than, 4 individuals and was maximized at pack size equal to 10.25 individuals. There was no relationship between annual *per capita* pack growth rates and population sizes (Table 3). There was a large number of group extinctions: 80% of the 20 studied packs went extinct over the study period, with vacant territory being either filled by new packs formed by bonding of opposite sexed groups, or by inclusion of this space into the territory of adjacent packs. Eighteen packs were formed throughout the study period. The number of packs formed each year was independent of population size but was positively related to the number of existing packs in the population (Table 3, Figure 3C, F). The size of a newly formed pack was independent of the population size at the year of its formation (χ^2^ = 0.76, *P* = 0.38, n = 18).

### Population performance: the lack of demographic Allee effect

We found that there was no relationship between *per capita* population growth rate and population size (Figure 3D). Removing the point at low population size that may be seen as an outlier did not affect the results. Moreover, pack sizes were independent of population sizes (χ^2^ = 2.27, *P* = 0.132, n = 46), showing that small and large packs coexist irrespective of population size (Figure 3E). The number of packs was correlated to the number of individuals in the population (R^2^ = 0.62), showing that a large population is comprised of more packs than in a smaller one (and not just larger packs, Figure 1B).

## Discussion

There is abundant evidence that amongst cooperative species group size is an important variable [43,44]. Furthermore, in many cases, including *Lycaon*, optimal group sizes have been identified [26,27,40,45]. However, the role of demographic Allee effects in this context has been neglected. Our consideration of Allee effects leads to the proposition that they operate at three different levels: the individual, the group and the population (component, group and demographic Allee effects). We thus introduce the concept of a group Allee effect, and its relevance for population dynamics and persistence.

We reveal how highly social species, suggested to be very susceptible to component Allee effects, can avoid the population extinction that is predicted to follow from Allee effects. In obligate cooperate breeders, individual fitness and group fate are highly dependent on each other, because individuals generally cannot survive outside groups. We propose that the organization of the population into a mix of large and small groups is indeed buffering (through the dispersal of large groups) the Allee effects occurring in small groups (at the individual and group levels). Furthermore, we highlight that it is indeed the evolution of eusociality and subsequent individual behavioural strategies (e.g. passive territoriality) that is facilitating the independence of groups and thus the co-existence of large and small groups in the populations, that compensates for the higher extinction risks of smaller packs caused by component and group Allee effects (we found 80% of groups went extinct in our *Lycaon* population, but the population persisted). If groups are independent, their sizes will also be independent of population size, so that the extinction of small groups will not be linked to the extinction of the population. We also hypothesized that, under these circumstances, density dependent effects at the levels of both individuals and groups will be determined by group size and not by population size. Our empirical analysis confirmed our hypotheses, demonstrating the existence of multiple component and group Allee effects in various life history traits in *Lycaon*, with frequent group extinctions, but no detectable consequences at the population level. We also found that Allee effect was caused by group sizes and not by population size, and that group size was independent of population size. This explains the seemingly contradictory results of previous studies on *Lycaon*, where both defendants and sceptics of Allee effects in this species had solid cases (see Table 1 and 2).

### Individual performance and the importance of group size

At the individual level, we found Allee effects in the breeding and survival of pups and dispersers, but the absence of an Allee effect related to the survival of either adults or yearlings was unexpected, although similar results have been found in other populations (Table 1 and 2). The component Allee effect affecting breeding could be explained by the scarcity of helpers in small packs. This is not a surprising result as the importance of helpers in this species has already been emphasized [26,40,45] and relationship between pack size and breeding success has also been shown in other populations [22,46] and see Table 1. The component Allee effect affecting the survival of pups could also be explained by its high dependence on the presence and abundance of helpers [22,27,33,47].

The dynamics of groups have significant bearing on the production of dispersers and success of colonization events [28]. We showed, for the first time, Allee effect in the survival of dispersers. Somers et al. [29] did not find such a relationship in the HiP population (which had been reintroduced after an absence of 50 years); they explained the lack of component Allee effects in terms of low interspecific competition and high prey availability. Here it is important to note that HiP was fenced which will unnaturally increase prey capture by reducing chase distances [48]. Consequently, unnaturally high energetic return from these factors and subsequent increased births [27] and survival could well negate Allee effects. Survival in our study was higher when dispersers came from a large pack and maximal when the packs from which they dispersed comprised more than 12 individuals. Higher survival of dispersers coming from larger packs probably resulted from a better body condition at dispersal than those dispersers coming from smaller packs, as individuals in larger packs bank more energy per day [27]. This was not mediated by the collective prowess of dispersers, as smaller packs did not produce smaller groups of dispersers in this population. There is a minimum quorum for dispersal so members of smaller packs delay dispersal until they can meet the minimum strength of numbers. However, perhaps because they were fitter as they would have banked more energy [27], dispersers from larger packs fared better, as has been suggested elsewhere [40,45].

### Group performance and the importance of group size

At the group level, we show that the number of pack formation events was positively related to the number of existing packs in the population. This agrees with results from the HiP population in South Africa [29], see Table 1. We also show that a pack’s growth rate depends on its size, and that it is positive when there are four or more individuals in the pack. This accords with previous studies that have demonstrated that a threshold in pack size exists; packs of more than four to six *Lycaon* faring better than did smaller packs [26,27]. Throughout our analyses, our results are consistent in showing an optimal group performance of 10–12 pack members (Figures 2C,F, 3F) and these data are in striking concordance with net rate of energetic intake data [27] which also show rapid individual and therefore group returns. These data highlight how at a population level where foraging returns are favourable, either naturally or unnaturally as in the case of fenced reserves, obligate co-operators such as *Lycaon* are perhaps better fitted than their competitors to exploit favourable conditions. Consequently, it is probable that it is the group Allee level effect that protects the species in leaner times long enough for rapid recovery where foraging and prey availability conditions are optimised.

### The lack of demographic Allee effect: low extinction risk at low numbers

At the population level, we found no evidence of a demographic Allee effect. This is consistent with seemingly paradoxical results from previous work in small populations of *Lycaon*, which not only failed to find it, but even showed negative density dependence of population growth rate [27,29,46]. As in previous published studies, our results on the demographic Allee effect are drawn from only one population, but this population varied greatly in size during ours long-term study. Future work might further test our hypothesis through a meta-analysis of all recently published long-term studies, profiting from their differing populations size and pack size ranges (see Table 1 and 2).

### Group independence benefits sociality

Here, absence of a demographic Allee effect seems to be related to the independence, that we demonstrated here, between group sizes and population sizes, and recently suggested by Woodroffe [32]. Previous literature reveals inconsistent results as to whether population decline is associated with a decrease in pack size or not. For example, MacLellan et al. [49] show that for ungulates declining populations were characterized by smaller group sizes. However, a lack of relationship between group size and population size has been seen in Serengeti lions, *Panthera leo*[50] and in populations of wild dogs in Kenya and Tanzania [32,45]. Somers et al. [29] showed a positive relationship between group and population sizes in *Lycaon* in South Africa, but the range of the population size and the number of packs analysed was smaller than in our study and than in Woodroffe [32]; in addition, the comparison may be inappropriate because the fences as well as the introduction of groups into the population in their study could have affected the composition of packs. Indeed, it has been shown that pre-release socialization and group fission following release is relatively frequent in wild dog reintroductions [51] what could have affected the size of the pack at release (as well as the corresponding estimations of yearly pack sizes) and processes such as pack formation or extinction.

The evidence of the empirical analysis suggests that relative independence amongst the dynamics of different groups has the consequence of preventing population extinctions at low numbers (thus, protecting populations from demographic Allee effects). Social groups seem to be self-contained, fluctuating in size independently of each other, in the sense that the fate of one particular group has practically no effect on the fate of other groups. This independence is strengthened by the fact that *Lycaon pictus* favours inter-group avoidance with large packs allowing smaller packs to utilize adjacent territories without harassment [52]. There appears to be a non-confrontational form of space-use where even large groups avoid areas recently hunted by smaller groups by use of scent [39,46], probably to save wasting energy attempting to capture predator-sensitized prey, or fights thus avoiding injuries that would be detrimental for both groups. This system occurs in several large carnivores that compete for territory; it is best described as “drifting territoriality” where pack ranges are sympatric over time, but parapatric at any point in time [52,53]. These results thus support our hypothesis that group sizes and fates are largely independent of each other.

Of course, alternative hypothesis could challenge our interpretation. For example, genetic effects (including genetic Allee effects) could be involved, although the time scale of this study suggests this is not a strong argument here. Similarly, it could be argued that smaller groups might decline faster than larger ones because animals of lower fitness are restricted to marginal habitat (in terms of food availability, prevalence of competitors/predators or risk of anthropogenic mortality). Interestingly this is not the case, as larger packs in this study did not exclude smaller packs from better quality territory. Despite the impossibility to test for the multidimensional facets of habitat quality in our very large study area, we remain confident that the combination of our logical argumentation, solid long-term data and previous publications of the crucial importance of group size for *Lycaon* fitness (Table 1 and 2) and on the lack of demographic Allee effects and pack-population size relationships in other populations [29-32], constitute a favourable set in support of our hypothesis.

## Conclusions

The evolutionary mechanisms that maintain cooperation differ qualitatively and quantitatively between different animal societies [17,43]. Here, we suggest that the architecture of a population comprising autonomous groups of different sizes and fates that are rather independent of the population size emerges from the particular structure, behaviour and dynamics of social groups. Kin selection and group augmentation hypotheses could both have favoured group living. However, selection would also favour mechanisms that compensate for the dependence on conspecifics associated with extreme sociality. Therefore, it is likely that the emergence of autonomous groups could have resulted in the absorption of the repercussions of component Allee effects. An emergent effect of such mechanisms could be to lower the risk of population extinction through the prevention of demographic Allee effects. Our findings for *Lycaon* are clear, and may be generally applicable to other obligate cooperatively breeding species. If so, the extinction risks caused by Allee effects have been amongst the selective pressures favouring individuals that organized themselves into relatively autonomous groups. In this way, the evolution of group living could have played a major role in the avoidance of extinctions threatened by Allee effects.

## Methods

### Field data

Data were collected between 1989 and 2002 in 6000 km2 of Hwange National Park, Zimbabwe and contiguous peripheral areas. Details of the population can be found elsewhere [54]. Individuals were identified using their unique coat markings, and followed throughout the year using both radio-tracking and opportunistic independent observations. Packs were defined as potentially reproductive groups containing at least an alpha pair (the breeding pair). Social status was determined by direct observation at dens, cover marking, and spatial proximity [27]. Radio-collared individuals and den watching was used only on packs that were deemed habituated to observer presence at this critical time. Pups were identified at emergence providing a record of the number of dogs in each class category at all times [26]. Three age classes were therefore distinguished: pups (<1 year), yearlings (≥1 year and <2 year) and adults (≥2 years). In the first year of the study of a pack the age classes were either determined from the fact that the life history of the individuals in the dispersal groups that formed the pack was already known (13 packs) or in the case of individuals whose history was unknown which included immigrants assessment was made by looking at dentition and scarring to get an age estimator. We defined yearly periods commencing with whelping in May/June, and finishing just before breeding the next year. Packs formed during the yearly period or extirpated before the end of the yearly periods (i.e., <12 months) were removed from the analyses to ensure unbiased demographic fates as they do not cover the totality of the yearly period. Annual pack sizes were defined as the total number of adults and yearlings of a pack at the end of each yearly period. Research on wild dogs followed internationally recognized guidelines; the Zimbawe government and the Hwange National Park give permits to carry out this study. Anaesthesia for collaring was authorised by the Zimbabwe Veterinary Association Under DDL 85-92.

### Testing for component Allee effects: reproduction and survival

In group-living species, traits of individual fitness could be related to group size and/or to population sizes. We thus tested the relationships between yearly pack sizes and population sizes with: (i) litter size, defined as the number of pups born each yearly period in each pack. Only one female bred in each pack; (ii) per capita productivity, defined as the number of pups reaching yearling status divided by pack size; (iii) annual survival of adults, yearlings and pups (we established survival rates in relation to the total number of individuals early in the year for each age class); (iv) survival of dispersers, defined as the number of dispersers that survived until the beginning of the next yearly period in relation to the total number of dispersers for each dispersal event. Dispersal survival was based on data from dispersing dogs that were fitted with radio-collars (40% of the studied individuals) and colored belts (10%). Female dispersers stayed close to their natal territory while males went as far as 570 km [55]. Dispersal events occurred throughout the year, preventing calculation of annual survival rates for dispersers.

### Testing for group Allee effects: group life spans and growth rates

Group fitness could be related to group size and/or to population size. We first assessed the potential relationship between pack lifespan (in months) and mean pack size (defined as the average of annual pack sizes for each pack). According to our predictions, if smaller packs have a shorter life span than larger packs this would be evidence of an Allee effect at the group level. Furthermore, a positive relationship between annual *per capita* pack growth rate and annual pack size and/or annual population size would reveal an Allee effect. We calculated an annual *per capita* pack growth rate as the relative increase in pack size (*N*) between two consecutive years (*t*) *N*_*t+1*_*/N*_*t*_[56]. Annual *per capita* pack growth rates are the net output of all the processes encompassed within pack dynamics including reproduction, survival and dispersal.

Finally, we examined whether an Allee effect was affecting pack formation in three ways: testing the relationship between the number of packs formed each year and the population size, between the number of packs formed each year and the number of existing packs, and between the size of the starting pack and the population size.

### Testing for demographic Allee effects: population growth rate

At the population level, we calculated the population size as the sum of the number of adults and yearlings at the start of the yearly period for all packs existing that year. We calculated annual *per capita* population growth rate as the relative increase in population size between two consecutive years (similarly to the annual *per capita* pack growth rate). We tested for a demographic Allee effect by examining the relationship between population size and *per capita* population growth rate. Although our data are drawn from only one population, that population varied in size so greatly during our long-term study that variation in *per capita* population growth rate can be used to test for demographic Allee effects. Finally, we explored the dependence between pack size and population size.

### Data analyses

Normality of all variables was verified and we used General Linear Models (SAS v.9.1, PROC GENMOD, [57]) to test relationships between each trait of fitness and pack or population size. Exceptions were survival rates, which were binomial variables (number of survivors in relation to the total initial number for each yearly period or dispersal event). In these cases, we used generalized linear models with binomial distribution and logit link function, the scale parameter was held fixed at one, and the lack of overdispersion was verified by looking that the deviance divided by its degrees of freedom was near one.

In some cases, data from packs were correlated within each pack; we handled this covariance structure by introducing the pack as a repeated measures variable (‘repeated subject’ SAS v.9.1, PROC GENMOD) in the analyses, because we had data of the same pack in consecutive years. This was the case for the analysis of component Allee effects in reproduction and survival, for analysis of Allee effects in the pack growth rate and for testing the relationship between pack sizes and population sizes. It was not the case for the analysis of pack creation, for the analysis of the pack life span because averages for each pack were used, nor was it the case for the analysis of the population growth rate because sums of pack numbers for each year were used. Repeated measures thus accounted for potential confounding effects of temporal variation in demographic parameters. Possible confounding effects of spatial variation in demographic parameters are reduced as packs stayed in much the same range throughout a given year. An Allee effect is demonstrated in each trait when it is positively related to density or size, for example through a simple linear, positive relationship [56]. However it is likely that negative density dependence also occurs at high densities and both effects can be tested simultaneously fitting a quadratic function of the form y = a + bx – cx^2^, where y is the fitness trait; x is pack or population size; a is a constant; b scales the linear term indicating an Allee effect when b > 0; and c scales the quadratic term capturing the curvilinear relationship that results from adding negative density dependence to Allee effects when c < 0. The quadratic fit was removed when it was not significant; thus final models could either contain both terms, only the linear term or no terms. Fitting a quadratic function allowed us to calculate the size that maximizes each fitness trait, which can be used as an estimate of the size below which Allee effects starts to dominate over negative density dependence [56].

## Competing interests

The authors declare that they have no competing interests.

## Authors’ contributions

EA designed research, performed the statistical analysis and drafted the manuscript. GR designed research, performed data collection and helped to draft the manuscript. FC and DM designed research and helped to draft the manuscript. All authors read and approved the final manuscript.

## Supplementary Material

Additional file 1 **Allee effects described in *Lycaon pictus *within the last decade. A**. Parameters in which Allee effects (AE) and pack-population sizes relationship have been described in wild dogs populations. **B**. Complete references and details on described wild dogs populations. Click here for file
