# Supplementary material for: Do social groups prevent Allee effect related extinctions?: The case of wild dogs
Source: Front Zool. 2013 Mar 15;10:11. doi: 10.1186/1742-9994-10-11 (PMC3626796; doi:10.1186/1742-9994-10-11)
Supplement: Additional file 1 — Allee effects described in Lycaon pictus within the last decade. A. Parameters in which Allee effects (AE) and pack-population sizes relationship have been described in wild dogs populations. B. Complete references and details on described wild dogs populations. [file 1742-9994-10-11-S1.pdf]

Elena Angulo, Greg S. A. Rasmussen, David W. Macdonald and Franck Courchamp
Corresponding author: angulo@ebd.csic.es, Estación Biológica de Doñana, CSIC, Avda. Americo Vespucio s/n, 41092 Sevilla, Spain

Additional File 1. A. Parameters in which Allee effects (AE) and pack-population sizes relationship have been described in *Lycaon pictus* within the last decade.

	Reference	Parameter	AE	YES/NO	Explanations given in the text
1	Courchamp et al. 2000	Demographic Allee effect	d	YES	The risk of population extinction is higher when modelling 9 pack population with Allee effects rather than direct density dependence.
		Pack extinction	g	YES	Higher rates of pack extinction when modelling population of 9 packs with Allee effects rather than direct density dependence.
		Pack formation	g	YES	Higher probability of failure to colonize territories by dispersers when modelling a 9 packs population with Allee effects rather than direct density dependence.
		Allee threshold in number of packs	d	YES	Population reaching a small number of packs (around 5) are likely to go completely extinct.
2	Courchamp & Macdonald 2001	Pup survival	c	YES	Small breeding groups are less successful in rearing young.
		Breeding - Litter size	c	YES	Small breeding groups are less successful in producing young.
		Hunting efficiency	c	YES	Ability to locate food, kill prey or overrule kleptoparasites decline in small foraging groups and can, in turn, reduce individual survival and/or fecundity.
3	Courchamp et al. 2002	Survival	c	YES	Less efficient in cooperative defence when fewer.
		Hunting efficiency	c	YES	Small packs performed an aditional hunt when they left a pup guard. This result confirms the prediction of a high costs of pup-guarding for small packs.
		Pup guarding	c	YES	Pup-guarding was significantly more likely in larger packs.
		Allee threshold in pack size	c	YES	A critical threshold at a size of about five individuals: more than 5 adults to feed and guard pups.
4	Creel & Creel 2002	Pup survival	c	YES	Comparing survival rates accross populations, pup survival relates positively to population density
		Pup, yearling and adult survival	c	NO	Some of the variation in survival is due to density, although the correlations are non-significant, but they are all strongly negative. Comparing survival rates accross populations, adult survival relates negatively to population density.
		Breeding - Litter size	c	YES	Packs of 10 adults or more produced litters twice as large as litters in packs with 9 adults or fewer.
		Pup survival	c	YES	Packs of 10 adults or more raised three times more yearlings than packs with 9 adults or fewer.
		Hunting success	c	YES	Hunting success (kills/hunt) increase and mean distance of succcessful chases decrease in larger packs (packs $\geq$ 10 adults).
		Hunting efficiency	c	YES	Mean mass of prey killed and mean number of prey killed simultaneously increased significantly as the number of adult wild dogs increased.
		Population size vs Pack size		YES	Pack size is not related to population density. (…) Mean pack size holds quite constant as density changes.
5	Creel et al. 2004	Pup survival	c	YES	The number of pups raised to one year increases as pack size increases.
		Breeding - Litter size	c	YES	The number of pups born increases as the number of adult pack-members increases.
6	Carbone et al. 2005	Hunting efficiency	c	YES	Kleptoprasitism from spotted hyena would not have a substantial affect on feeding performance for large groups of wild dogs. However, kleptoparasitism could have a substantial impact on declining populations with small group sizes of wild dogs.
7	Buettner et al. 2007	Juvenile survival	c	YES	Pup survival to the age of 9 and 12 months was significantly positively correlated with pack size.
		Breeding - Litter size	c	YES	Number of pups emerging from the den was positively related to pack size.
		Breeding success	c	NO	No evidence of reproductive failure in small packs.
8	McNutt et al. 2008	Pup survival	c	YES	Larger packs raised significantly more surviving pups.
		Breeding - Litter size	c	YES	Females living in large packs produced significantly more pups.
9	Rasmussen et al. 2008	Breeding - Litter size	c	YES	Smaller packs have a lower reproductive output (significantly smaller litters).
		Hunting success	c	NO	No relationship between pack size and the likelihood of success once a chase had been initiated.
		Energy balance	c	YES	Positive relationship between pack size and the net rate of energy intake up to a pack size of 10.
		Pack extinction	g	YES	Small packs are energetically compromised. With reduction in group size resulting in lower net foraging gains and smaller litters and eventually in group extirpation.

Additional File 1 (cont). A. Parameters in which Allee effects (AE) and pack-population sizes relationship have been described in *Lycaon pictus* within the last decade.

	Reference	Parameter	AE	YES/NO	Explanations given in the text
10	Somers et al. 2008	Demographic AE	d	NO	Population growth rate was significantly negatively related to population size.
		Pup, yearling and adult survival	c	NO	No component Allee effects at the pack level (litter size, number of pups raised to 1 year, annual survival rates for pups, yearlings and adults).
		Survival of dispersers	c	NO	Pack size did not influence annual survival rates of dispersers.
		Disperser group size	c	NO	Pack size did not influence disperser group size.
		Breeding -Litter size	c	NO	No component Allee effects at the pack level explained by low interspecific competition and high prey availability.
		Pack formation	g	YES	The annual number of pack formation events was positively related to the number of existing packs.
		Population size vs Pack size		YES	Population size was more positively related to the number of packs than to pack size.
11	Gusset & Macdonald 2010	Pup, yearling, adult survival	c	NO	Pack size did not have a significant effect on number of pups surviving to one year of age, pup, yearling or adult survival effect.
		Breeding -Litter size	c	YES	Pack size had a significantly positive effect on litter size
12	Woodroffe 2011	Demographic AE	d	NO	A marginally nonsignificant trend suggested that the rate of population growth might have been related negatively to population size.
		Pup survival, survival all	c	NO	Although small packs raised fewer pups than did larger packs, even the smallest packs successfully raised some pups and thus increased in size
		Breeding -Litter size	c	YES	Larger packs produced larger litters
		Breeding success	c	NO	Breeding failure did not appeare more frequent in smaller packs.
		Pack extinction	g	NO	Pack extinction did not appeare more frequent in smaller packs.
		Allee threshold in pack size	g	NO	No evidence was found of a critical pack size below which either reproduction or pack survival was compromised.
		Population size vs Pack size		NO	Because population density increased over time, subsequent analyses use time as a proxy for density (…) No significant effect of pack size occurred over time.
13	Our study	Demographic AE	d	NO	No relationship between per capita population growth rate and population size.
		Pup survival	c	YES	Pup survival was lower in smaller packs and higher at medium pack sizes.
		Adult & Juvenile survival	c	NO	Absence of an Allee effect related to the survival of either adults or yearlings.
		Dispersers survival	c	YES	Positive density dependence in the survival of dispersers.
		Disperser group	c	NO	Larger packs did not produce larger groups of dispersers in this population
		Breeding -Litter size	c	YES	Litter size was positively and significantly correlated with pack size, showing an Allee effect related to reproduction at lower pack sizes
		Per capita productivity	c	YES	Smaller packs had lower productivity than did packs of medium sizes. Using the estimated quadratic fit, the pack size that maximized the per capita productivity was 10.20 individuals.
		Pack creation	g	YES	The number of pack formed was positively related to the number of existing packs but was independent of population size. The size of the starting pack was also independent of population size
		Pack life spam	g	YES	There was a significant, positive relationship between mean pack size and pack life span: smaller packs had the shortest life span.
		Pack growth rate	g	YES	There was a quadratic, significant relationship between annual pack size and annual per capita pack growth rates (...). We estimated that pack growth rate was positive at pack size equal to, or higher than, 4 individuals and was maximized at pack size equal to 10.25 individuals.
		Pack size Allee threshold	g	YES	At the group level, we show that a pack’s growth rate depends on its size, and that it is positive when there are four or more individuals in the pack
		Pack size optimum	g	YES	Throughout our analyses, our results are consistent in showing an optimal group performance of 10-12 pack members
		Population size vs Pack Size		NO	Pack sizes were independent of population sizes, showing that small and large packs coexist irrespective of population size. The number of packs was correlated to the number of individuals in the population, showing that a large population is comprised of more packs than in a smaller one.

Note: Before the year 2000, literature on the effects of pack size is reviewed by Courchamp & Macdonald 2001, Table 1.

Additional File 1 (cont). B. Complete references and details on described wild dogs populations

Ref	Country	Location	Total area (Km2)	Date range	Density range	Number of packs	Pack size range	Pack mean	Trend along study
3	Z	Hwange National Park		5 years					
4	T	Selous Game Reserve	2600	1991-1996	A: 48-63 (40) B: 880		C: 3-20	8,9 ad	S
5	SA	Kruger National Park	4280	1989-2003	A: 19-39	A: 8 to 12	A: 2-36	10,4	
	T	Selous Game Reserve	2600	1991-1997	A: 35-46 (38)	7	A: 2-24 or 2-52	8,9 ad or 18,9 ad+yy+pups	
	B	Northen Botswana	2600<br>TOT: 9480		A: 5 to 35, B: 700-850	A: 6 to 13	A: 2-30	10,4 ad+yy	
6	T	Serengueti		1964-1987	B: 0-100				D
7	SA	Kruger Nat Park		1989-2004		B: 30			
8	B		3000	15 years	C: 700-986	B: 78-88	C: 2-30	10.4	
9	Z	Hwange National Park	5500	8 years			B: 0-21		I
10	SA	Hluhluwe-iMfolozi Park		1980-2004	A: 0,3-3,4 (1.6)<br>B: 3-31 (14.1)		A: 2-24	8.1	Ri
11	SA		380	1995-2006		C: 1.7	A: 2-17	6.2	Ri
12	K			2000-2008	B: 10-200 (core area)<br>B: 50-290 (all study area)		A: 3-21		Rc
13	Z	Hwange National Park	6000	1989-2002	D: 7-53 (22)	A: 1 to 6			I

Country: B: Botswana; K: Kenya; SA: South Africa; T: Tanzania; Z: Zimbabwe.
Density range: A: indiv/1000km2, B: individuals, C: adults, D: Individuals/year (mean in parenthesis)
Number of packs: A: per year, B: total number of packs studied, C: Mean pack number
Pack size range: A: individuals, B: adults + yealrings, C: adults
Population trend: D: decreasing to extinction, I: increasing, Ri: reintroduced; Rc: recolonization from absence along 20 years; S: Stable

References

1 Courchamp F, Clutton-Brock T, Grenfell B (2000) Multipack dynamics and the Allee effect in the African wild dog, Lycaon pictus. Anim Conserv 3:277-285

2 Courchamp F, Macdonald DW (2001) Crucial importance of pack size in the African wild dog Lycaon pictus. Anim Conserv 4:169-174

3 Courchamp F, Rasmussen GSA, Macdonald DW (2002) Small pack size imposes a trade-off between hunting and pup-guarding in the painted hunting dog Lycaon pictus. Behav Ecol 13:20-27

4 Creel S, Creel NM (2002) The African wild dog: behavior, ecology, and conservation. Princeton University Press, New Jersey

5 Creel S, McNutt JW, Mills MGL (2004) Demography and Population Dynamics of African Wild Dogs in Three Critical Populations. In Biology and Conservation of Wild Canids, ed by DW Macdonald & C Sillero-Zubiri, Oxford University Press, pp. 337-350.

6 Carbone C, Frame L, Frame G, Malcolm J, Fanshawe J, FitzGibbon C, Schaller G, Gordon IJ, Rowcliffe JM, Du Toit JT (2005) Feeding success of African wild dogs (Lycaon pictus) in the Serengeti : the effects of group size and kleptoparasitism. J. Zool. 266:153-161

7 Bluettner UK, Davies-Mostert HT, du Toit JT, Mills MGL (2007) Factors affecting juvenile survival in African wild dogs (Lycaon pictus) in Kruger National Park, South Africa. J Zool: 272:10-19

8 McNutt JW, Silk JB (2008) Pup production, sex ratios, and survivorship in African wild dos, Lycaon pictus. Behav Ecol Sociobiol 62:1061-1067

9 Rasmussen GSA, Gusset M, Courchamp F, Macdonald DW (2008) Achilles heel of sociality revealed by energetic poverty trap in cursorial hunters. Am Nat 172:508-518

10 Somers MJ, Graf JA, Szykman M, Slotow R, Gusset M (2008) Dynamics of a small re-introduced population of wild dogs over 25 years: Allee effects and the implications of sociality for endangered species' recovery. Oecol 158:239-247

11 Gusset M, Macdonald DW (2010) Group size effects in cooperatively breeding African wild dogs. Anim Behav 79:425-428

12 Woodroffe R. (2011) Demography of a recovering African wild dog (Lycaon pictus) population. J Mammal 92: 305-315.

13 Our study
